# The importance of social factors in the association between physical activity and depression in children

**DOI:** 10.1186/s13034-020-00335-5

**Published:** 2020-06-27

**Authors:** May I. Conley, Isabella Hindley, Arielle Baskin-Sommers, Dylan G. Gee, B. J. Casey, Monica D. Rosenberg

**Affiliations:** 1 Department of Psychology, Yale University, 2 Hillhouse Avenue, New Haven, CT 06520 USA; 2 Department of Psychology, The University of Chicago, Chicago, IL USA

**Keywords:** Childhood, Depression, Development, Physical activity, Friendships

## Abstract

**Background:**

Physical activity is associated with reduced depression in youth and adults. However, our understanding of how different aspects of youth activities—specifically, the degree to which they are social, team-oriented, and physical—relate to mental health in children is less clear.

**Methods:**

Here we use a data-driven approach to characterize the degree to which physical and non-physical youth activities are social and team-oriented. We then examine the relationship between depressive symptoms and participation in different clusters of youth activities using mixed effect models and causal mediation analyses in 11,875 children from the Adolescent Brain Cognitive Development (ABCD) Study. We test our hypotheses in an original sample (*n *= 4520, NDA release 1.1) and replication sample of participants (*n* = 7355, NDA release 2.0.1).

**Results:**

We show and replicate that social–physical activities are associated with lower depressive symptoms. Next, we demonstrate that social connections, measured by number of close friends, partially mediate the association between social–physical activities and lower depressive symptoms.

**Conclusions:**

Our results provide a rubric for using data-driven techniques to investigate different aspects of youth activities and highlight the social dynamics of physical activities as a possible protective factor against depression in childhood.

## Background

The U.S. Department of Health and Human Services has linked physical activity to improved health and reduced risk for long-term disease in youth, adults, and older adults [75]. Physical activity, generally, is associated with decreases in symptoms of depression and anxiety in adults [5, 21, 34, 66], increases in neurocognitive performance in older adults [48], and improved mental health and self-esteem in children and adolescents [11, 16, 35, 42, 46, 49, 71, 76]. Moreover, physical activity related to participation in team sports may be particularly beneficial for mental health and depression [17, 24, 35], suggesting that different aspects of activities might distinctly impact mental health. Although previous work has characterized differences in childhood depression related to team and individual sports [35], no research, to date, specifically examines the relationship between other aspects of activities (e.g., physical or non-physical; social or non-social) and depressive symptoms in children.

The prevalence of internalizing disorders in youth underscores the importance of identifying protective factors that may inform prevention and intervention [20, 51]. Nearly 20% of youth aged 13–18 already have experienced major depression [3] and over 30% receive anxiety disorder diagnoses [45]. Among young people who receive diagnoses, fewer than half receive treatment [57], often after symptoms have become severe enough to warrant intervention. Furthermore, internalizing diagnoses are likely underreported [72] and the prevalence of youth internalizing disorders may, therefore, be understated. Although efforts to develop early detection instruments will be key to providing precise care for youth, understanding the impact of various childhood experiences on protecting against internalizing symptoms is a critical step forward.

Late childhood and early adolescence is not only a time when a number of mental health problems begin to emerge [45], but also a time when parents, educators, and policymakers scrutinize over which activities will lead to the healthiest outcomes for young people. Although participation in organized activities is believed to be important for boosting well-being and adjustment in youth [25], research on the optimal number and type of activities for mental health yields mixed findings. Overall diversity, or breadth, of activities has been linked to positive adjustment in adolescents and young adults [29]. However, specific outcomes vary by activity type, with research consistently showing that sport participation may have long-term mental health benefits [11, 42, 70]. Among individuals with a history of adverse childhood experiences, team-sport participation in adolescence has been related to increased resilience against later internalizing disorders in adulthood [24]. Physical activity also has been associated with improvement in depressive symptoms in adolescents aged 13–17 [16]. While less is known about whether younger children receive the same benefit, recent work analyzing data from over 4000 children across the US (a subset of the data included in this study) reported that team sport participation was associated with lower levels of depressive symptoms, specifically in male children [35, 36]. Taken together, previous work suggests that physical activities can help mitigate depressive symptoms in youth.

Like physical activity, social factors can be protective against depression and anxiety during childhood and adolescence [38]. Youth who experience loneliness or peer rejection are at increased risk for depression in later adolescence and adulthood [13, 63, 64]. Additionally, quality of friendships has been shown to predict youth behavior with positive relationships leading to favorable outcomes and negative relationships increasing risk for poor outcomes [52].

Importantly, however, physical activity and social factors are not necessarily independent. Rather, many youth activities involve both physical and social components, and social connections such as close friendships, potentially forged and maintained in these contexts [26], may explain some of the protective effects of physical activities on depressive symptoms. For example, Babiss and Gangwisch [4] showed that participation in sports may lead to decreases in harmful behaviors and increases in perceived social support and self-esteem in adolescents. Similarly, the presence of prosocial peers has been found to mediate the relationship between time spent in sports and depression in high school students [28]. In contrast, participation in social *non*-*physical* activities such as performing arts can increase feelings of social isolation over time [8], indicating that the influence of social connections on mental health outcomes may not be equal across all activities [14]. To date, no studies have systematically disentangled the relative benefits of social aspects of physical activity as they relate to depressive symptoms in youth. In other words, it remains unclear whether physical activity itself is protective, or whether the social aspect of some physical activities account for some or all of their benefits for children’s mental health. Accordingly, researchers have used different approaches to capture and categorize youth activities.

Prior work broadly examining youth activities suggests that person-centered approaches, which evaluate unique profiles of individuals’ overall activity participation but do not explicitly test social aspects, provide a more holistic account of how young people spend their time [7, 27]. Different activities have distinct social and emotional demands [12, 50], suggesting that person-centered approaches may be improved by explicitly examining the social aspects of youth activities while examining individuals’ unique profiles of activity participation. Though important, investigations of the social components of youth activities are sparsely represented in the literature, partially because prior research relies on measures that do not explicitly investigate social- and non-social factors [30]. One reason for the paucity of research on social components of youth activities may be previous methodological constraints. However, recent advances in technology such as Amazon’s Mechanical Turk (MTurk) provide reliable and efficient online platforms for collecting psychological and other data [60, 65]. MTurk offers a flexible, easy-to-implement method for “crowdsourcing” opinions [15], such as those about the dynamics of social- and non-social youth activities.

### Current studies

Here, we investigate the relationship between different types of youth activities and depressive symptoms. In the first study, we assess the degree to which different physical and non-physical youth activities are perceived as being social and team-oriented in an adult sample using MTurk. In the second study, we evaluate associations between caregiver-reports of youth activities assessed with the Sports Activities Involvement Questionnaire (SAIQ) and youth internalizing symptoms assessed with the Child Behavior Checklist (CBCL) using data from the first release (NIMH Data Archive (NDA) Release 1.1, 10.15154/1412097) of Adolescent Brain Cognitive Development℠ Study (ABCD Study^®^) data (*n *= 4520 children ages 9 and 10). In particular, we focus on depressive symptoms, given previous research showing an association between physical activity and depressive symptomatology [17, 24, 35]. Next, we assess the strength of any observed effects by testing them in the second data release (NDA Release 2.0, 10.15154/1503209) of ABCD data (*n* = 7355 children ages 9 and 10) for replication. Finally, we evaluate whether engagement in social–physical activities is related to increases in social connections (e.g., close friendships). In supplementary analyses, we test whether physical activity is related to depressive symptoms over and above other internalizing symptoms (e.g., somatic, anxious). Given previous work showing that team sports, specifically, are associated with the largest improvements in mental health, our overarching hypotheses are that: (1) participation in social–physical activities, but not other types of activities, will be associated with lower depressive symptoms; and (2) social connections, in general, will mediate the relationship between social–physical activities and lower depressive symptoms.

### Study 1: data-driven categorization of youth activities

Although previous work has examined physical activity as a protective factor against depression [16], the social aspects of youth activities have not been systematically evaluated. In Study 1, we examine how two samples of adults characterize youth activities reported on the ABCD Sports and Activities Involvement Questionnaire (SAIQ; described below), a parent-report measure. To evaluate the social- and team-dimensions of SAIQ activities, we collected Qualtrics survey data using Amazon’s MTurk.

## Methods

### Sample

Two U.S. samples (*n*_*1*_ = 249, *n*_*2*_=251) were collected on MTurk. We restricted our sample to adults who had more than a 75% Human Intelligence Task (HIT; the term for tasks completed on MTurk) approval rate and who had completed more than 50 HITs. All data were removed from 159 participants who had intra-rater reliability scores less than *r* = 0.50 or who were suspected repeat raters (determined by evaluating IP address in conjunction with provided demographic data). The final MTurk sample included 341 participants (*n*_*1*_ = 163, *n*_*2*_=178; 147 female, 194 male) who had a mean age of 30.1 (range = 18-40; SD = 4.67) and self-identified as Asian (*n* = 27), Black/African American (*n *= 36), White/Caucasian (*n* = 248), Hispanic or Latinx (*n* = 23), and Other (*n* = 7). All participants provided consent in accordance with procedures approved by the institutional review board at Yale University and were paid for their participation. These data are available as supplementary materials [see Additional file 2].

### Measures

#### The ABCD Sports Activities Involvement Questionnaire (SAIQ)

The SAIQ is a caregiver-report survey developed for the ABCD study that evaluates lifetime history, frequency and duration, and past-year participation in sports, non-sport activities, and other hobbies [6]. As detailed in Barch et al. [6], the SAIQ is modeled after the Vermont Health and Behavioral Questionnaire and the Dutch Health Behavioral Questionnaire [41].

### Procedure

Data for Study 1 were collected on MTurk using surveys constructed in Qualtrics. The Qualtrics survey included 3 parts: an initial activity rating, an intervening cognitive task to prevent participants from immediately re-rating activities [19], and a second activity rating used to generate reliability scores to ensure participant adherence. Since there are multiple ways to classify the sociality of youth activities, each rating survey asked participants to score every SAIQ activity on two dimensions: team to individual and social to non-social.

Participants were presented with two sliding bar scales for each activity: one asking *“how team*- *or individually*-*oriented”* each activity is from 0.0 (completely team-oriented) to 10.0 (completely individual) and another asking *“how social”* each activity is on a scale from 0.0 (completely social) to 10.0 (not social at all). Team-oriented was defined as “*an activity that is always done in a group”* and individual was defined as *“an activity that is not team*-*oriented at all, and is always completed solo.*” Participants were also given an example for the social-dimension stating, *“For example, if you were asked about “hanging out with friends,” you might rate it a “0,” indicating that it is completely social*.” After each survey component, participants received a 6-digit code that was pasted into a box in MTurk to authorize their completion. Because some of the individual questions on the SAIQ survey group more than one activity together (Table 1; e.g., swimming and water polo are grouped as a single activity) [35], activities were further divided into 35 separate items.

**Table 1:** Categorization of SAIQ activities based on results of the Amazon Mechanical Turk (MTurk) survey

Social physical	Non-social physical	Social non-physical	Non-social non-physical
Softball, Baseball	Dance, ballet	Drama, theater, acting, film	Musical Instrument (Singing, Choir, Guitar, Piano, Drums, Violin, Flute, Band, Rock Band, Orchestra)
Basketball	Climbing	Competitive games like chess, cards, or darts	Drawing, Painting, Graphic Art, Photography, Pottery, Sculpting
Field Hockey	Gymnastics		Crafts like Knitting, Building Model Cars of Airplanes
Football	Horse-riding, polo		Hobbies like collecting stamps or coins
Ice Hockey	Ice Skating		
Lacrosse	Inline Skating		
Rugby	Martial Arts		
Soccer	Skateboarding		
Waterpolo, Swimming	Snowboarding, Skiing		
Volleyball	Surfing		
	Tennis		
	Track, running, cross-country		
	Wrestling		
	Yoga, Tai Chi		

### Analytic approach

#### Reliability of MTurk youth activity categorization

Rating reliability was calculated at the individual and group levels. For each participant, reliability was assessed by calculating the Pearson correlation between the first and second ratings of each activity. Next, mean ratings for each activity were calculated for both MTurk samples. Pearson’s *r* and intraclass correlation coefficients (ICC) were generated to evaluate reliability and consistency within and across the two independent samples.

#### Clustering youth activities

Data obtained in both MTurk samples was sufficiently consistent within (*r*_(1)_= 0.99, *r*_(2)_= 0.99; ICC_(1)_ = 0.99; ICC_(2)_ = 0.99) and across samples (e.g., comparing rating 1 and 2; *r*_(1,2)_= 0.99; ICC_(1,2)_ = 0.99), so data from both ratings from all 341 participants were used to generate mean team–individual and social–non-social scores for each youth activity. Next, we assigned a binary physical or non-physical score to each activity (e.g., football and climbing were categorized as *physical* whereas drawing and painting were categorized as *non*-*physical*) [40].

Activities were clustered into data-driven categories based on their ratings along the three dimensions: team–individual, social–non-social, and physical–non-physical. Specifically, ratings were used to generate distance measures for k-means cluster analysis using the cluster and factorextra packages in R [44, 53]. The optimal cluster size and number of activity clusters (*k*) were determined by using the elbow method (Additional file 1: Fig. S1a) and cross-referenced by computing silhouette scores (Additional file 1: Fig. S1b) [47]. Because our primary question was whether different *aspects* of youth activities (as opposed to different activities themselves) were related to depressive symptoms, we used this clustering method rather than relating depressive symptoms to continuous measures of social and team-orientation.

### Study 1 results: MTurk youth activities categorization

**Fig. 1:**
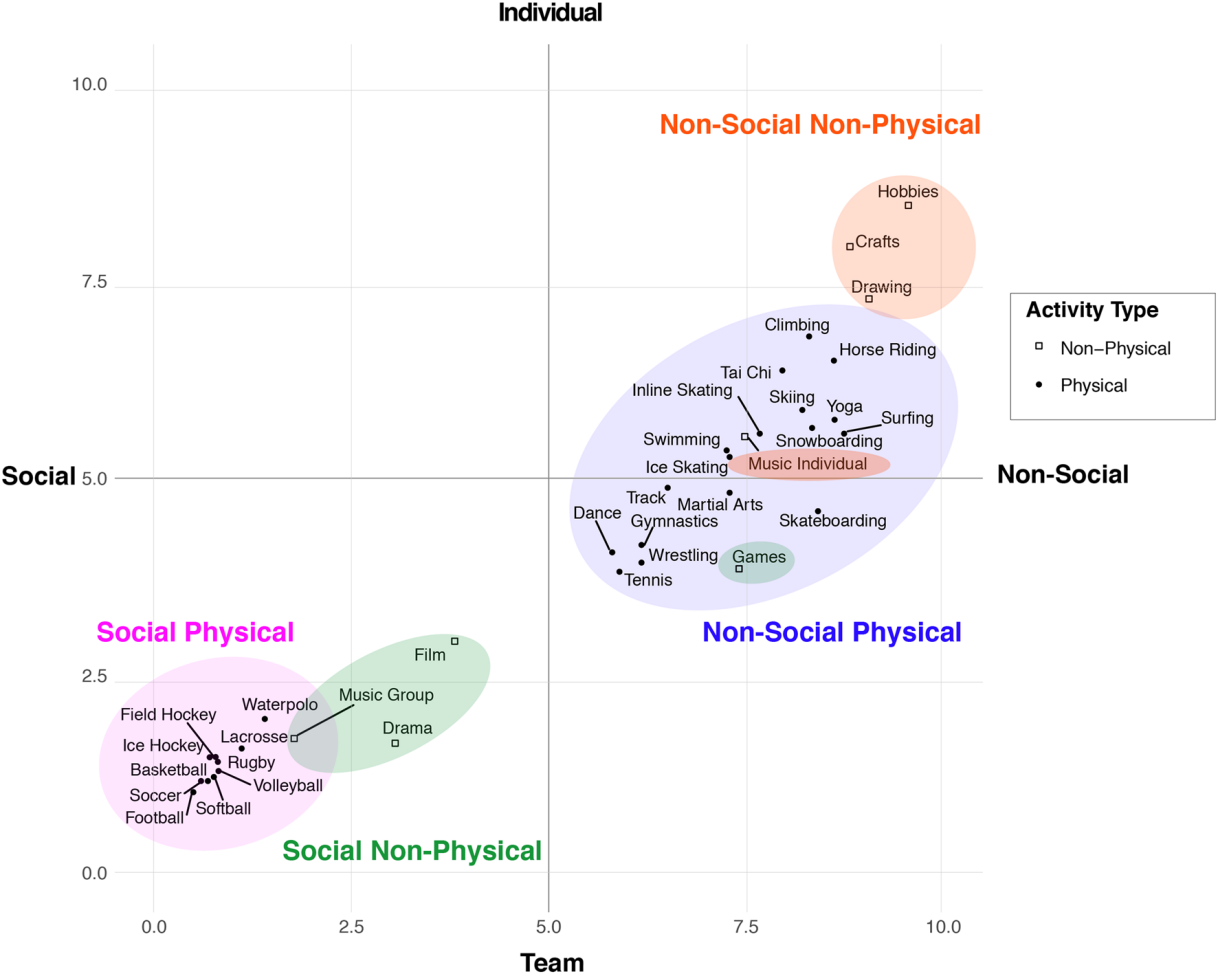
Quadrant plot of MTurk Youth Activities Survey showing results of *k*-means clustering of SAIQ activities. Social–non-social and team–individual dimensions are visualized across the x- and y-axes, respectively, and the physical–non-physical dimension is visualized with shading and bullet-shape

To evaluate how each activity from the SAIQ related to the team–individual and social–non-social MTurk rating dimensions and the physical–non-physical dimension, *n*-means clustering was used to extract *k* data-driven categories from all MTurk ratings (*n *= 500 observations). For simplicity, the 4 *k*-means derived clusters were named based on the social and physical dimensions. However, the *k*-means clustering analysis was performed using all three dimensions defined above (i.e., physical–non-physical, social–non-social, and team–individual). Physicality of each activity is visualized with shading and bullet-shape in Fig. 1. Because the location of the bend in the plot comparing total within-cluster sum of squares (wss) to different values of *k* (Additional file 1: Fig. S1a) was less distinct (e.g., gradual decline from *k *= 3 to *k* = 4 clusters), average silhouette method [47] results (*k* = 4) were used (Additional file 1: Fig. S1b). The final *k*-means analysis was performed extracting results using 4 clusters: social–physical, non-social–physical, social–non-physical, and non-social–non-physical. Further descriptions of each activity included in these categories are outlined in Fig. 1 and Table 1.

### Study 1 summary

The motivation for Study 1 was to use a data-driven approach to examine how adults characterize social aspects of youth activities from the SAIQ (a parent-report measure from the ABCD battery). Specifically, we evaluated the degree to which different physical and non-physical youth activities are perceived as being social and team-oriented using MTurk. Using *k*-means cluster analysis, we extracted 4 distinct clusters, suggesting that youth activities can reliably be categorized by their degree of sociality and physicality. The results of Study 1 serve as a rubric for classifying social- and physical-aspects of youth activities. All online data collected are available for use in the scientific community and made available in (see Additional file 2).

## Study 2: youth activities and depression in the ABCD Study

### Methods

#### Sample

Participants included 11,875 9 and 10 year old children in the ABCD Study (https://ABCDStudy.org, https://nda.nih.gov/abcd) [77]. The ABCD sample was collected using a school-based recruitment method to comprise a geographically, demographically, and socioeconomically diverse sample of children and families across 21 sites in the United States [18, 31]. Primary hypotheses were tested in the original sample including 4520 children (47.5% Female; 19.6% Hispanic/Latino, 9.8% Black, 2.3% Asian, 9.4% Other; mean age in months = 118.29 (*SD *= 7.48), range = 108-131) from the first curated release of ABCD data (NDA Release 1.1, 10.15154/1412097). Replication of findings was then tested in a replication sample which consisted of 7355 new children (i.e., children not included in ABCD Release 1.1; 48.0% Female; 20.7% Hispanic/Latino, 18.1% Black, 2.1% Asian, 10.4% Other; mean age in months = 120.01 (*SD* = 7.29), range = 108–131) from a subsequent release of ABCD data (NDA Release 2.0.1, 10.15154/1503209). Exclusionary criteria for this study include a diagnosis of autism spectrum disorder, history of epilepsy or seizures, and incomplete CBCL, demographic or SAIQ data.

#### Measures

The ABCD study assesses physical and mental health, culture and environment, neurocognition, neuroimaging and biospecimens that are harmonized across sites. Data are publicly available through NDA (https://ABCDStudy.org, https://nda.nih.gov/abcd). For this study we used data from the Child Behavior Checklist (CBCL), SAIQ (described above) and ABCD Other Resilience Scale (called YSR in ABCD release notes) detailed below.

#### Child Behavior Checklist (CBCL)

The Achenbach System of Empirically Based Assessment CBCL is a widely-used, dimensional parent-report assessment for identifying problem behavior in children that is standardized and normed by sex, age, informant, and ethnicity (internal consistency [Cronbach’s alpha] = 0.90 and reliability [Pearson’s *r*] = 0.72 for internalizing disorders) [1]. The CBCL contains 120 items that are rated and summed into eight syndrome scales and two broad-band scale scores (internalizing and externalizing). Internalizing includes three subscales: depressive (withdrawn), anxiety and somatic complaints. Externalizing includes two subscales: aggressive behavior and rule-breaking tendencies. Our primary dependent variable was depressive symptoms (i.e., withdrawn/depressive CBCL subscale). Supplementary analyses explore associations of other subscales of internalizing disorders.

#### Social connections: the ABCD Other Resilience Scale (YSR)

The ABCD YSR is a child-report survey that measures number of and type of youth’s friendships (e.g., general or close; https://nda.nih.gov/abcd). Data from 2 YSR (abcd_ysr01) items was summed for each participant:

1. resiliency5b_y: How many CLOSE friends that are boys do you have?
2. resiliency6b_y: How many CLOSE friends that are girls do you have?

Before responding to these two items, participants are told that close friendships are characterized by having fun, enjoying spending time together, and trust.

#### Youth activity participation

Data from the 29 SAIQ activities were used to evaluate how many different types of activities children participated in over the past year based on results from the study 1 described above. All SAIQ activities are visualized in Fig. 1 and detailed in Table 1. All participants were assigned a count score for the number of activities they participated in across each of the four *k*-means-derived clusters of activities: social–physical, non-social–physical, social–non-physical, and non-social–non-physical. For example, participating in basketball and soccer would equate to a social–physical score of 2. Individuals who did not participate in any type of activity were included in all analyses and assigned a score of “0” in each category.

### Analytic approach

#### Depressive symptoms and youth activities

**Fig. 2:**
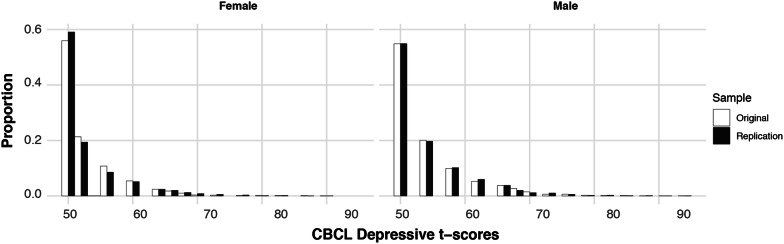
Bar chart showing proportions of CBCL t-scores for depressive subscale across original (*n *= 4393) and replication samples (*n* = 7127) by sex

Descriptive statistics were calculated for depressive symptoms derived from the CBCL depressive/withdrawn subscale in each sample and across activity categories. Because the CBCL subscale symptom t-score distributions were positively skewed (Fig. 2), the data were log transformed to approximate normality. To further examine differences across activity types, we ran linear mixed effects models, which are robust to assumptions of non-normality that are characteristic of large sample sizes [33, 58], using the nlme package in R [61]. All models covaried for sex, age in months, age-squared, household income, race, the interaction of sex and age, and the interaction of sex and age-squared. We included age-squared to capture non-linear effects of age that may relate to dynamic changes occurring during this period of development [37]. The ABCD sample includes siblings and twins, so data collection site and family nested within site were included as random effects in all models. Additionally, significance was evaluated using permutation testing to lessen the risk of detecting significance due to large sample size only (original sample *n* = 4520; replication sample *n *= 7355). Nonparametric significance was assessed by using the permute function in R (Simpson [69]) randomly shuffling data 1000 times to generate null distributions to compare main effects against. Effect sizes were generated using the MuMIn package in R and both marginal *r*^2^ (i.e., variance explained by fixed effects) and conditional *r*^2^ (i.e., variance explained by both fixed and random effects) are reported [9]. To examine the variance due to each category of activity separately, change in *r*^2^ ($\Delta$*r*^2^) was generated by calculating the difference in marginal *r*^2^ between our models and null models [59].

We conducted a primary analysis to evaluate how frequency of participation (i.e., the sum of a child’s total activity participation over the past 12 months) in each data-driven activity cluster related to depressive symptoms. Next, to evaluate whether the association with youth activities was specific to depressive symptoms, or whether relationships were explained by associations with internalizing symptoms more generally, we tested observed effects on depressive symptoms when controlling for anxious and somatic symptoms. All analyses were Bonferroni corrected for multiple comparisons and tested for replication in a separate sample.

#### Youth activities, social factors, and depressive symptoms

Causal mediation analyses were used to evaluate whether: (a) social–physical activities were associated with overall number of close friends; and if (b) increased number of close friends was associated with lower depressive symptoms [61]. Lastly, we tested whether number of close friends mediated the association between social–physical activities and lower depressive symptoms using the mediation package in R [73]. The average causal mediation effect (ACME) and average direct effect (ADE) were generated to estimate indirect and direct effects of social connections on depressive symptoms. Both estimates (ACME and ADE) were generated using non-parametric bootstrapping (5000 iterations, *p *< 0.05). All mediation analyses were run using Poisson regressions and race, age, sex, income, site, and family were included as covariates.

### Study 2 results: youth activities and depression in the ABCD Study

**Fig. 3:**
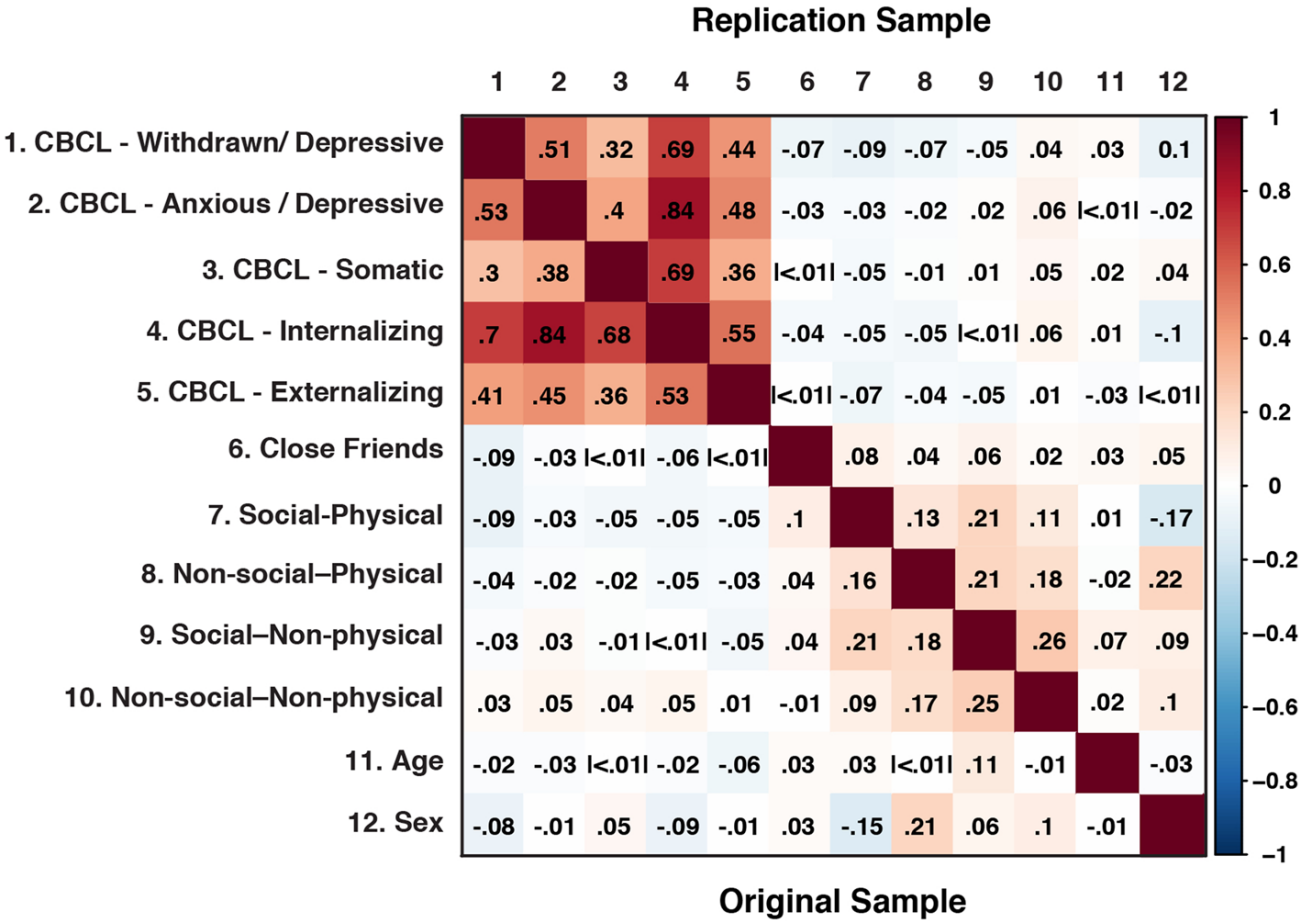
First order correlations (Spearman’s *rho*) between CBCL symptoms, close friends, activity types, age, and sex (original sample below diagonal [Correlations across samples were not generated to preserve the use of the replication sample as an independent sample]; replication sample above diagonal). Spearman correlations are represented in color as indicated on the scale on the right (i.e., positive correlations in warm colors and negative correlations in cool colors; sex was dummy-coded with 0 = male, 1 = female)

The mean depressive symptom t-score was 53.47 (*SD* = 5.70) in the original sample and 53.48 (*SD* = 5.82) in the replication sample (Fig. 2). To examine first-order associations between CBCL symptoms and activity types, first-order correlations (Spearman’s *rho*) were examined and are visualized in Fig. 3. Consistent with previous research Gorham et al. [35], overall depressive scores within the original sample were higher in males (mean = 54.16, *SD* = 6.30) than females (mean = 52.73; *SD* = 4.86) (*t* = 8.40, df = 4255.9, *p* < 0.001). This pattern also was identified in the replication sample, with higher depressive scores in males (mean = 54.13, *SD *= 6.25) than in females (mean = 52.80, *SD *= 5.24) (*t *= 9.78, df = 7019, *p *< 0.001) (see Additional file 1: Section 1 for further analyses by sex). Additional descriptive statistics for all internalizing subscales are provided in Additional file 1: Table S1a, b and Additional file 1: Figs. S2, S3.

**Fig. 4:**
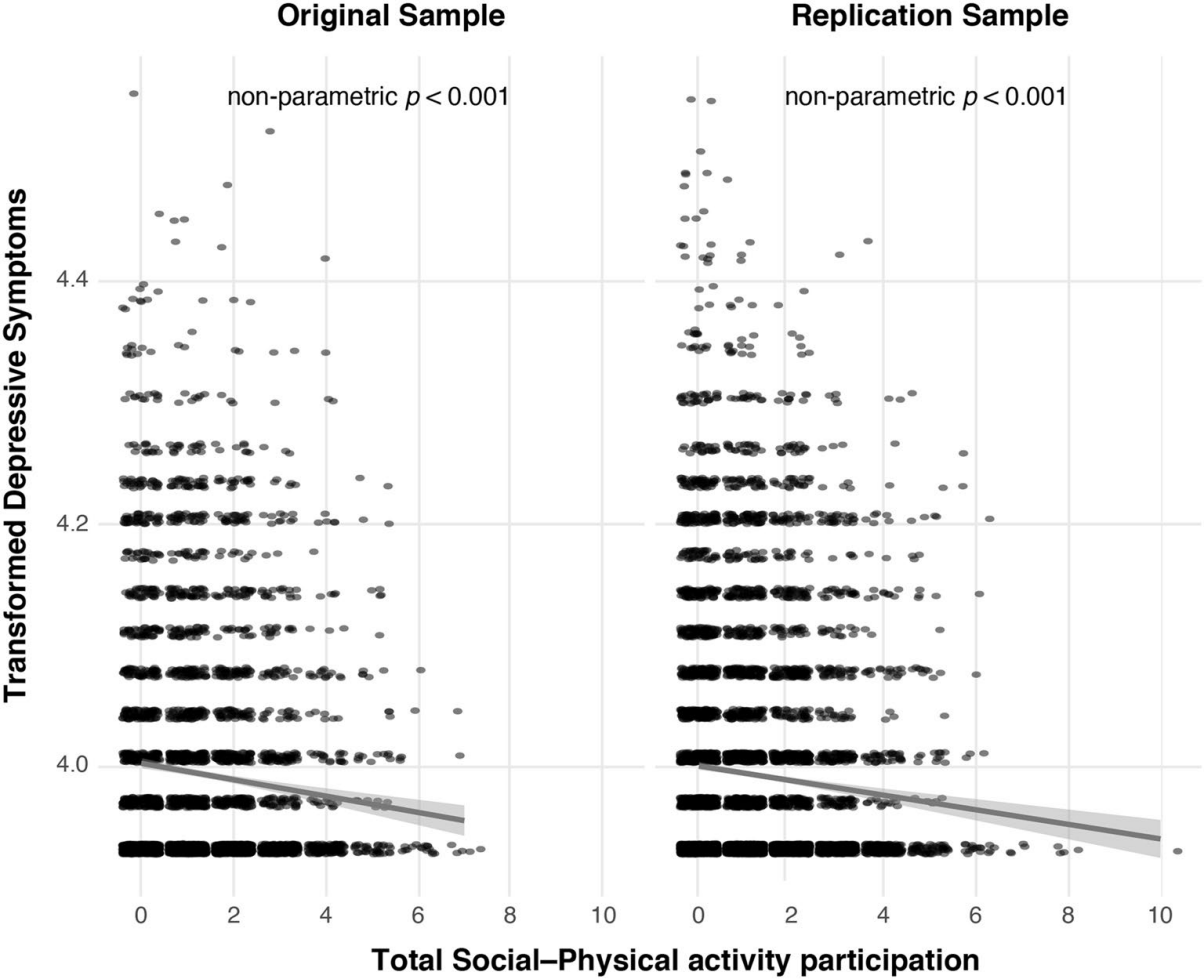
Scatter plot of log-transformed depressive symptoms by participation in social–physical activities. Linear mixed effect models with permutation testing were used to examine the differences in symptoms according to total social–physical activity participation in the past 12 months

**Fig. 5:**
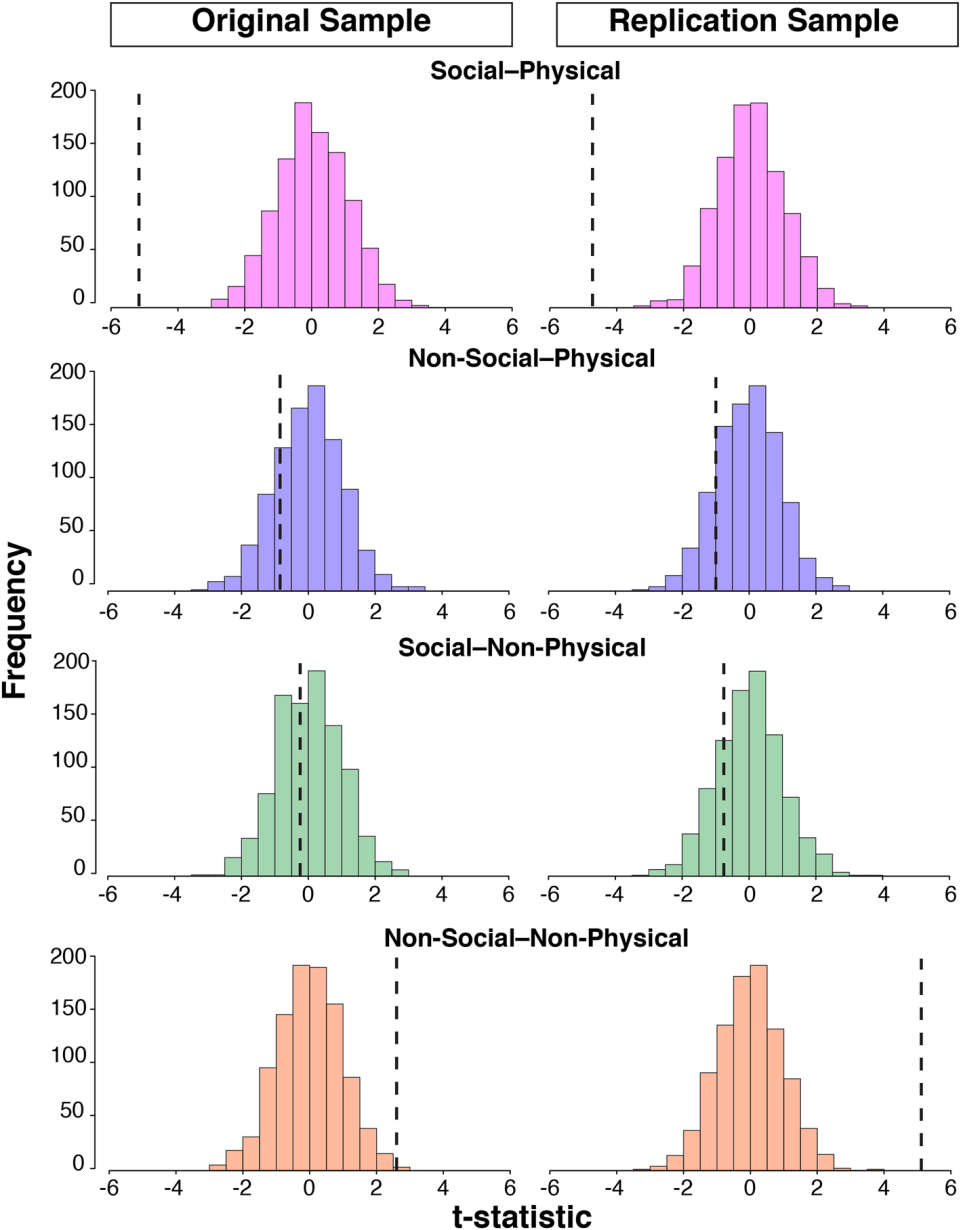
Histograms showing replication of the associations between participation in unique clusters of activities and depressive symptoms, based on the results of the permutation testing (The histograms show the null distribution of t-statistics generated by randomly shuffling data 1,000 times. Observed effects in the tails of or outside the null distribution demonstrate that effects are non-parametrically significant and more extreme than chance). The dashed line indicates the observed t-statistic derived from linear mixed effect models. The negative t-statistic observed for social–physical activities is expected because of the negative association between social–physical activities and depression

Consistent with our hypothesis, a mixed effect model revealed a significant association between higher participation in unique social–physical activities and lower depressive symptom scores in the original sample ($\beta$_1_ = − 0.01 (*SE *= 0.001), *p* < 0.001, df = 548; $\Delta$*r*^2^ = 0.01) and replication sample ($\beta$_2_ = − 0.01 (*SE *= 0.001), *p* < 0.001, df = 847;$\Delta$*r*^2^ = 0.01) (Fig. 4 and Table 2). Because of the large original sample *n* = 4520 and replication sample *n *= 7355, permutation testing was used to evaluate significance. Results of non-parametric significance testing show that observed effects are more extreme than effects observed in any null model (Fig. 5, top row). Analyses by sex showed that there was an association between activity participation and depressive symptoms in both males and females separately (see Additional file 1: Section 1 and Additional file 1: Fig. S4).

**Table 2:** Relationship between involvement in activities on log transformed depressive symptoms

	Original	Replication
Intercept	3.74^***^ (0.66)	3.98^***^ (0.51)
Social–physical	− 0.01^***^ (0.001)	− 0.01^***^ (0.001)
Non-social–physical	− 0.003 (0.003)	− 0.001 (0.002)
Social–non-physical	− 0.0004 (0.002)	− 0.002 (0.002)
Non-social–non-physical	0.01^**^ (0.002)	0.01^***^ (0.002)
Sex	1.39 (0.90)	− 0.34 (0.71)
> $100 K	− 0.02^***^ (0.01)	− 0.05^***^ (0.004)
$25–50 K	0.01 (0.01)	− 0.02^***^ (0.004)
$50–100 K	− 0.01^**^(0.01)	− 0.03^***^ (0.004)
White	0.01 (0.005)	0.004 (0.004)
Black	− 0.01^*^ (0.01)	− 0.01^**^ (0.004)
Asian	− 0.01 (0.01)	− 0.01 (0.01)
Other	0.01 (0.01)	0.01 (0.005)
Age	0.004 (0.01)	0.0003 (0.01)
Age^2^	− 0.0000 (0.0000)	− 0.0000 (0.0000)
Sex:age	− 0.02 (0.02)	0.01 (0.01)
Sex:age^2^	0.0001 (0.0001)	− 0.0000 (0.0001)
Marginal r^2^	0.04	0.05
Conditional r^2^	0.38	0.22
Observations	4151	6652
Log Likelihood	3891.46	6130.23
Akaike Inf. Crit.	− 7742.91	− 12,220.47
Bayesian Inf. Crit.	− 7616.37	− 12,084.47

All analyses include site and family nested within site as random effects

Reporting $$\beta$$ (SE) *p < 0.05**p < 0.01***p < 0.001

Because we were interested in how different types of youth activities relate to depressive symptoms, we included the 3 other MTurk-derived activity categories in our models. There was no significant association between non-social–physical activity participation and depressive symptoms in the original ($\beta_{1}$ = 0.003 (*SE *= 0.003), *p* = 0.32, df = 548; $\Delta$*r*^2^ = 0.001) or replication sample ($\beta$_2_ = − 0.001 (*SE *= 0.002), *p* = 0.52, df = 847; $\Delta$*r*^2^ = 0.003) (Fig. 5, second row). Similarly, social–non-physical activity participation was not related to symptoms in the original ($\beta$_1_ = − 0.0004 (*SE *= 0.002), *p* = 0.87, df = 548; $\Delta$*r*^2^ = 0.001) or replication sample ($\beta$_2_ = − 0.002 (*SE *= 0.002), *p* = 0.23, df = 847; $\Delta$*r*^2^ = 0.003) (Fig. 5, third row). Interestingly, non-social–non-physical activities were associated with increased depressive symptoms in the original ($\beta$_1_ = 0.01 (*SE *= 0.002), *p* < 0.01, df = 548; $\Delta$*r*^2^ = 0.002) and replication samples ($\beta$_2_ = 0.01 (*SE *= 0.002), *p* < 0.001, df = 847;$\Delta$*r*^2^ = 0.01) (Fig. 5, bottom row). These findings further support our hypothesis that social–physical activities are uniquely associated with lower depressive symptoms in childhood.

#### Robustness of youth activity participation to depressive symptoms

We conducted an additional analysis on depressive symptoms when controlling for anxious and somatic symptoms in order to test whether youth activity participation relates specifically to depressive symptoms over and above these other internalizing symptoms. Consistent with our hypotheses and primary findings, social–physical activities predicted lower depression symptoms when controlling for anxiety and somatic symptoms in the original ($\beta$_1_ = − 0.01 (*SE *= 0.001), *p *< 0.001, *df *= 523) and replication sample ($\beta$_2_ = − 0.003 (*SE *= 0.001), *p *< 0.001, *df *= 796). We provide results from supplementary analyses within the CBCL subscales (anxious and somatic) (Additional file 1: Fig. S2 and Additional file 1: Table S2) and broadband scales (internalizing and externalizing) (Additional file 1: Fig. S3 and Additional file 1: Table S3) in Additional file 1: Section 2 for comparison.

#### Mediation by social connections

**Fig. 6:**
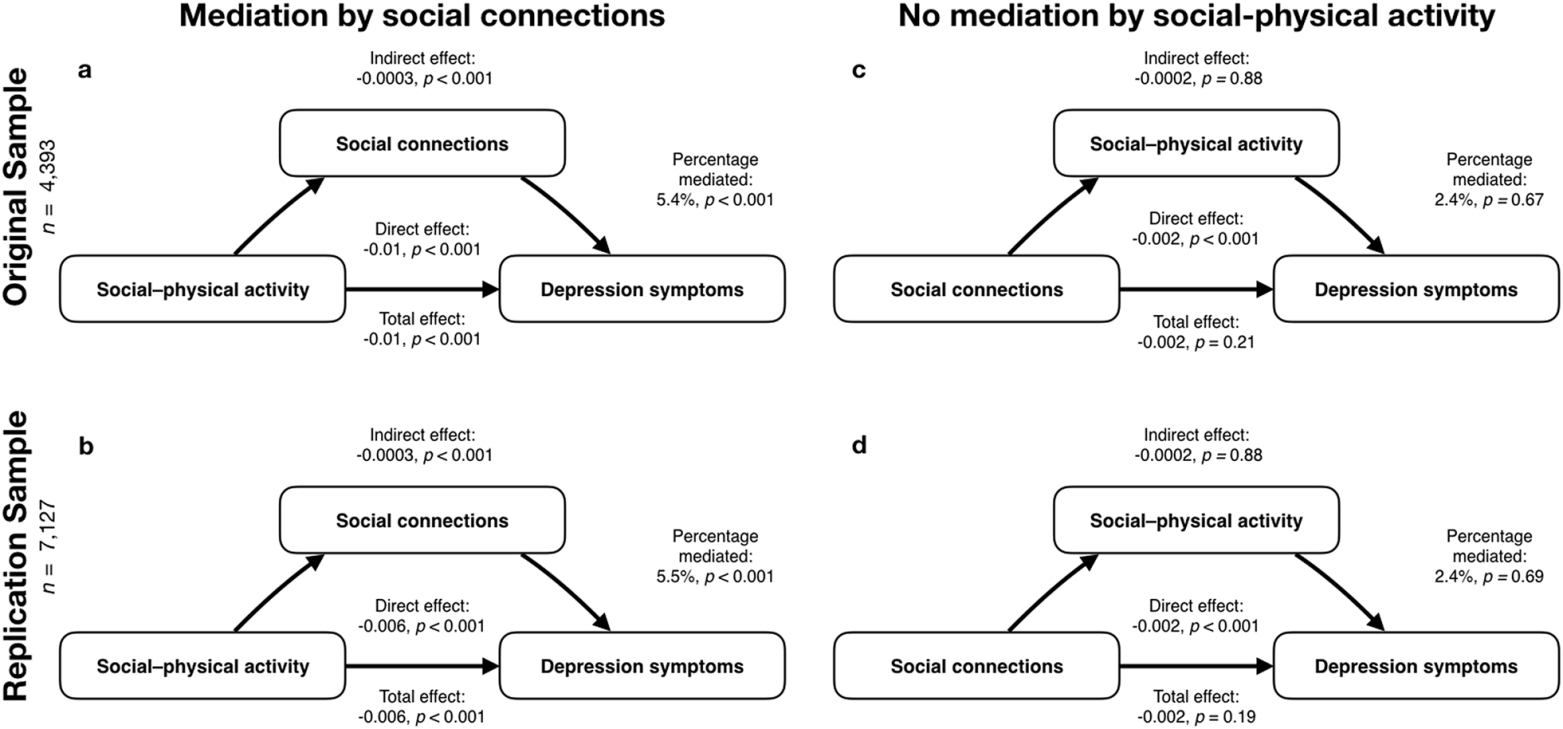
Visualization of models used to test mediation by social connections in original (**a**) and replication samples (**b**). Results from the supplementary reverse model are provided for comparison in original (**c**) and replication sample (**d**)

The primary aims of this study were to evaluate whether (1) social aspects of physical activities are particularly important in protecting against depression in youth and (2) social connections, as measured by number of close friends, explain some of the protective effects provided by social–physical activities. A causal mediation analysis showed that overall number of close friends partially mediated 5.4% of the influence of social–physical activity participation on depressive symptoms (ACME (indirect effect) = − 0.0003, 95% CI [− 0.001, − 0.0001], *p* < 0.001; ADE (direct effect) = − 0.01, 95% CI − 0.008, − 0.003], *p *< 0.001; total effect = − 0.01, 95% CI [− 0.008, − 0.004], *p* < 0.001; proportion mediated = 0.0542, 95% CI [0.02, 0.11], *p *< 0.001) in the original sample (Fig. 6a). These effects replicated with social connections partially mediating 5.5% of the effect of social–physical activity participation on depressive symptoms (ACME (indirect effect) = − 0.0003, 95% CI [− 0.001, − 0.0001], *p* < 0.001; ADE (direct effect) = − 0.006, 95% CI − 0.008, -0.003], *p *< 0.001 total effect = − 0.006, 95% CI [− 0.009, − 0.004], proportion mediated = 0.0549, 95% CI [0.02, 0.11]) in the replication sample (Fig. 6b). Specificity analyses were conducted and are visualized in Fig. 5c, d and reported in detail in Additional file 1: Section 3.

### Study 2 summary

The primary aim of Study 2 was to evaluate associations between clusters of youth activities and depressive symptoms in children. We also tested whether engagement in social–physical activities was related to increases in social connections, in general. We hypothesized that social connections, measured as number of close friends, would mediate the relationship between social–physical activities and lower depression. Using data from two separate samples of ABCD data, we show that participation in social–physical activities, but not other types of activities, is associated with lower depressive symptoms. Additionally, social connections partially mediated the relationship between social–physical activities and lower depressive symptoms highlighting a novel pathway that may provide insight into how participation in different types of activities relate to mental health outcomes in youth.

## Discussion

Physical activity is important for building healthy habits and protecting against mental and physical health problems. The primary goal of these studies was to use a data-driven classification method to investigate different aspects of youth activities that relate to depression in childhood, specifically focusing on social factors. We established that different types of youth activities can be reliably categorized on the degree to which they are physical and social/team-oriented. Building on previous work showing positive associations between team sports and mental health [16, 23, 24, 35, 42], we show that social–physical activities may be one protective factor against depressive symptoms in children. Furthermore, close friendships partially mediated the relationship between participation in social–physical activities and depressive symptoms. This finding highlights a novel pathway that provides insight into how social–physical activity participation may specifically protect against depression in children.

Previous work has identified that the social aspect of physical activity is important for maintaining activity participation [39]. Our results indicate that the social aspect of physical activities may present a unique opportunity for forging close friendships in childhood that ultimately increase perceived belongingness and other factors that protect against depression [10, 62, 67]. In contrast, non-social–non–physical activities were found to be associated with higher depressive symptoms. Taken together, these findings suggest that participation in any type of youth activity may not provide the same benefits or protect against the same risks. It is possible that non-social–non-physical activities may increase feelings of isolation in childhood, while social–physical activities may boost opportunities for close friendship and peer-support. These results demonstrate that social–physical activities may provide additional benefits above and beyond those of other types of youth activities. Loss of social connections has been linked to depression during adolescence and emerging adulthood [56]; therefore, getting children engaged in social–physical activity at an early age may be important for parents, educators, policymakers, and mental health professionals to consider. As depression is a form of psychopathology that can be resistant to both pharmacological and cognitive interventions [22, 74], and in light of other work showing that interventions such as cognitive behavioral therapy can be reinforced by exercise programs [55, 68], our results further highlight and specify one factor that may be important to target at an early age. Additionally, depression has been found to predict physical health status [32, 43] and physical activity levels [54], so encouraging youth to participate in social–physical activities may promote long-term positive outcomes and prove useful in bolstering healthy development across multiple domains.

The current findings build on previous work demonstrating that youth activities are one feature of children’s lives that are important for mental health outcomes, yet several limitations must be considered. First, while our analyses utilized a data-driven empirical method for categorizing youth activities, our study was constrained by the activities assessed on the SAIQ, which fails to include some popular youth activities (e.g., riding bikes). Second, parent/caregiver-report was used for estimating clinical symptoms and activity involvement. Although the CBCL is widely used with strong validity and reliability [1], past work suggests that it may be difficult for parents to identify internalizing symptoms particularly in childhood [72]. Future work may be enhanced by including youth-report measures of both internalizing symptoms and activity participation, in addition to utilizing more precise measures of physical activity such as FitBits or other pedometer technology. Third, our findings vary from recent work showing that team-sports are associated with lower depression in only males [35]. This discrepancy may be driven by different approaches in the measurement of youth activities (e.g., varying definitions of sports versus physicality) and differences in exclusionary criteria. For example, 328 additional subjects were removed from Gorham et al.’s [35] analyses due to missing or unusable brain data. Similarly, Gorham et al. [35] used categories that are not mutually exclusive, whereas in our analyses activities were sorted into one of four categories. Overall, we believe our results complement Gorham et al. [35] and both studies provide important information for future research about youth activities and mental health. Fourth, while non-parametrically significant, the overall magnitude of the association between social–physical activity and depression is small. This effect, however, replicated across independent samples, and significance was evaluated with permutation testing. Thus, while small, the relationship between social–physical activities and symptoms of depression appears robust, reliable, and replicable. Furthermore, we tested the effects of activity participation on depressive symptoms in parsimonious models that included all categories of activity at the same time, a conservative approach previously used by Fredricks and Eccles [29]. Taken together, although our results suggest social–physical activity participation is associated with lower depression in children, additional research is needed to characterize the real-world effects of interventions based on social–physical-activity for depression in youth.

The observed mediation effect—that close friendships partially mediate the relationship between social–physical activity participation and depressive symptoms—fills a gap in the literature about team-sports and depressive symptoms in youth. However, these analyses are limited in their ability to determine directionality. Recent work suggests a unidirectional protective relationship between physical activity and major depression in adults [76], but future work poised to assess directionality between activity participation and mental health throughout development is needed. For example, a core feature of several internalizing disorders is self-selecting out of various activities [2], and it is reasonable to expect that anxious or depressed youth may choose not to participate in activities (e.g., due to lower levels of motivation or social phobia). Our specificity analysis, detailed in Additional file 1: Section 3, shows that social–physical activities do not mediate the association between social connections and depression. This result suggests a distinct pathway whereby participation in social–physical activities may lead to an increased number of close friends, which in turn affects depression. Although we used a crowd sourcing approach to collect ratings of the social- and team-dynamics of youth activities, it is also possible that not all youth have the same experiences of social dynamics within any one activity. Future work may be improved by including youth-report measures rather than parent-report measures of the social-dynamics they perceive during various activities. The current study design does not allow us to determine causality; however, forthcoming releases of ABCD longitudinal data will allow us to evaluate how associations between youth activities and mental health change throughout development.

## Conclusions

In sum, results from the present studies indicate that social connections are an important contributor to known associations between participation in social–physical activities and lower depressive symptoms in 9- and 10-year-old children. The results from Study 1 provide researchers with a new rubric for evaluating different aspects of youth activities. The results from Study 2 serve as a baseline characterization of youth activities for future longitudinal follow-up of outcomes as risk for mental health problems increases in later childhood and adolescence. Overall, participation in social–physical activities may provide a target to unify efforts addressing physical and mental health goals.

## Supplementary information

**Additional file 1: Section 1.** Sex differences in relationship between social physical activities and depression. **Section 2.** Youth activities and other mental health symptoms. **Section 3.** Specificity analyses of mediation effects**. Figure S1a.** Line plot comparing total intra-cluster variation (total within sum of squares) to hypothetical number of clusters (*k*). **Figure S1b.** Line plot comparing average silhouette of observations for hypothetical number of clusters (*k*). **Figure S2.** Histogram of CBCL t-scores for subscale symptoms across original (*n* = 4,393) and replication samples (*n* = 7,127). **Figure S3.** Histogram of CBCL t-scores for broadband symptoms across original (*n* = 4,393) and replication samples (*n* = 7,127). **Figure S4** Scatter plot of transformed depressive symptoms by participation in social–physical activities split by sex. A mixed effect model was used to examine the differences in symptoms according to activity participation in the past 12 months. **Table S1a**. Descriptive statistics for original sample internalizing t-scores across all activity categories. **Table S1b**. Descriptive statistics for replication sample internalizing t-scores across all activity categories. **Table S2**. Relationship between involvement in clusters of SAIQ activities on normalized CBCL internalizing subscale symptoms. All analyses include site and family nested within site as random effects. **Table S3**. Relationship between involvement in clusters of SAIQ activities on CBCL broadband symptoms (t-scores). All analyses include site and family nested within site as random effects.

**Additional file 2.** Raw Qualtrics survey data for social- and team-dimesions of SAIQ activities.

## Data Availability

Data used in the preparation of this article were obtained from the Adolescent Brain Cognitive Development (ABCD) Study (https://abcdstudy.org), held in the NIMH Data Archive (NDA). This is a multisite, longitudinal study designed to recruit more than 10,000 children age 9–10 and follow them over 10 years into early adulthood. The ABCD Study is supported by the National Institutes of Health and additional federal partners under aware numbers *U01DA041022, U01DA041028, U01DA041048, U01DA041089, U01DA041106, U01DA041117, U01DA041120, U01DA041134, U01DA041148, U01DA041156, U01DA041174, U01DA041123, U01DA041147*. A full list of supporters is available at https://abdstudy.org/nih-collaborators. ABCD consortium investigators designed and implemented the study and/or provided data but did not necessarily participate in analysis or writing of this report. This manuscript reflects the views of the authors and may not reflect opinions or views of the NIH or ABCD consortium investigators. The ABCD data repository grows and changes over time. The ABCD data used in this report came from NIMH Data Archive Digital Object Identifier (DOI) 10.15154/1412097 (NDA Release 1.1) and 10.15154/1503209 (NDA Release 2.0). DOIs can be found at https://ndar.nih.gov/study.html?id=576 (NDA Release 1.1) and https://nda.nih.gov/study.html?id=634 (NDA Release 2.0). The dataset supporting the conclusions of Study 1 are included within the article (and Additional file 2).

## Abbreviations

ABCD: Adolescent Brain Cognitive Development Study
SAIQ: Sports Activities Involvement Questionnaire
CBCL: Child Behavior Checklist
NDA: NIMH Data Archive
