# Supplementary material for: The importance of social factors in the association between physical activity and depression in children
Source: Child Adolesc Psychiatry Ment Health. 2020 Jun 27;14:28. doi: 10.1186/s13034-020-00335-5 (PMC7321548; doi:10.1186/s13034-020-00335-5)
Supplement: Supplementary file 1 — Additional file 1: Section 1. Sex differences in relationship between social physical activities and depression. Section 2. Youth activities and other mental health symptoms. Section 3. Specificity analyses of mediation effects. Figure S1a. Line plot comparing total intra-cluster variation (total within sum of squares) to hypothetical number of clusters (k). Figure S1b. Line plot comparing average silhouette of observations for hypothetical number of clusters (k). Figure S2. Histogram of CBCL t-scores for subscale symptoms across original (n = 4,393) and replication samples (n = 7,127). Figure S3. Histogram of CBCL t-scores for broadband symptoms across original (n = 4,393) and replication samples (n = 7,127). Figure S4 Scatter plot of transformed depressive symptoms by participation in social–physical activities split by sex. A mixed effect model was used to examine the differences in symptoms according to activity participation in the past 12 months. Table S1a. Descriptive statistics for original sample internalizing t-scores across all activity categories. Table S1b. Descriptive statistics for replication sample internalizing t-scores across all activity categories. Table S2. Relationship between involvement in clusters of SAIQ activities on normalized CBCL internalizing subscale symptoms. All analyses include site and family nested within site as random effects. Table S3. Relationship between involvement in clusters of SAIQ activities on CBCL broadband symptoms (t-scores). All analyses include site and family nested within site as random effects. [file 13034_2020_335_MOESM1_ESM.docx]

Additional Materials

**Section 1: Sex differences in relationship between social physical activities and depression**

In light of recent findings by Gorham et al. (2019) showing that greater sports involvement was associated with lower depression in boys, but not girls, sex differences in frequency response were examined. We repeated our primary analyses separately by sex and found a negative association between participation in social-physical activities and depressive symptoms in males ($\beta$_1_ = -0.01 (*SE* = 0.002), *p* < 0.001*,* df = 205) and females ($\beta$_1_ = -0.003 (*SE* = 0.002), *p* < 0.01*,* df = 227) in the original sample. This pattern replicated with participation in social-physical activities associated with lower depressive symptoms in males ($\beta$_1_ = -0.01 (*SE* = 0.001), *p* < 0.001*,* df = 288) and females ($\beta$_1_ = -0.003 (*SE* = 0.002), *p* < 0.05*,* df = 227) (**Additional Figure 4**). As these associations were consistent across sex within each sample (*z*_1_ = -1.66, two-tailed *p* = 0.10; *z*_2_ = -1.70, two-tailed *p* = 0.09), we chose to include sex as a covariate in our primary analyses presented in the main text. Further attention to sex will be relevant throughout the developmental window and later adolescence when sex differences in internalizing disorders become more pronounced (Nolen-Hoeksema and Girgus, 2004; Martel, 2013).

**Section 2: Youth activities and other mental health symptoms**

Additional analyses within the two other internalizing subscales (anxious and somatic) (**Additional Figure 2**) and within the broadband scales (internalizing and externalizing) (**Additional** **Figure 3)** were conducted to demonstrate the robustness of observed effects to depressive symptoms. No association between participation in social-physical activities and anxiety symptoms was observed in the original sample ($\beta$_1_ *=* -0.001 (*SE* = 0.001), *p* = 0.13, *df* = 525). However, youth who engaged in more social-physical activities had lower anxiety in the replication sample ($\beta$_2_ *=* -0.002 (*SE* = 0.001), *p* < 0.05, *df* = 798). Similarly, there was no relationship between participation in social-physical activities and somatic symptoms ($\beta$_1_ *=* -0.01 (*SE* = 0.01), *p* = 0.06, *df* = 523; $\beta$_2_ *=* -0.002 (*SE* = 0.01), *p* = 0.06, *df* = 796) or externalizing symptoms ($\beta$_1_ *=* 0.13 (*SE* = 0.13), *p* = 0.44, *df* = 523; $\beta$_2_ *=* -0.11 (*SE* = 0.11), *p* = 0.12, *df* = 798) in either sample. These results are provided in **Additional Table 2** and **Additional** **Table 3**.

**Section 3: Specificity analyses of mediation effects**

Specificity of observed effects was evaluated by testing the reverse order of the mediator models featured in our main text (**Figure 6, main text**) A second mediation analysis showed that participation in social-physical activities did not mediate the relationship between social connections and depressive symptoms in the original sample (ACME (indirect effect) = -0.0002, 95% CI = [-0.003, 0.003], *p* = 0.88; ADE (direct effect) = -0.002, 95% CI = -0.003, -0.0007], *p* <0.001; total effect =-0.002, 95% CI = [-0.005, 0.001], *p* = 0.21; proportion mediated = 0.239, 95% CI = [-7.22, 6.37], *p* = 0.67). These effects also were observed in the replication sample with no mediation effect of social-physical activity on social connections and depressive symptoms (ACME (indirect effect) = -0.0002, 95% CI = [-0.003, 0.003], *p* = 0.88; ADE (direct effect) = -0.002, 95% CI = -0.003, -0.0007], *p* <0.001; total effect =-0.002, 95% CI = [-0.005, 0.001], *p* = 0. 19; proportion mediated = 0.237, 95% CI = [-7.22, 6.94], *p* = 0.69). Although the design of the current study precludes examining temporal order between variables, these results suggest specificity of the effect of social connections partially mediating the association between increased social–physical activity and lower depressive symptoms.


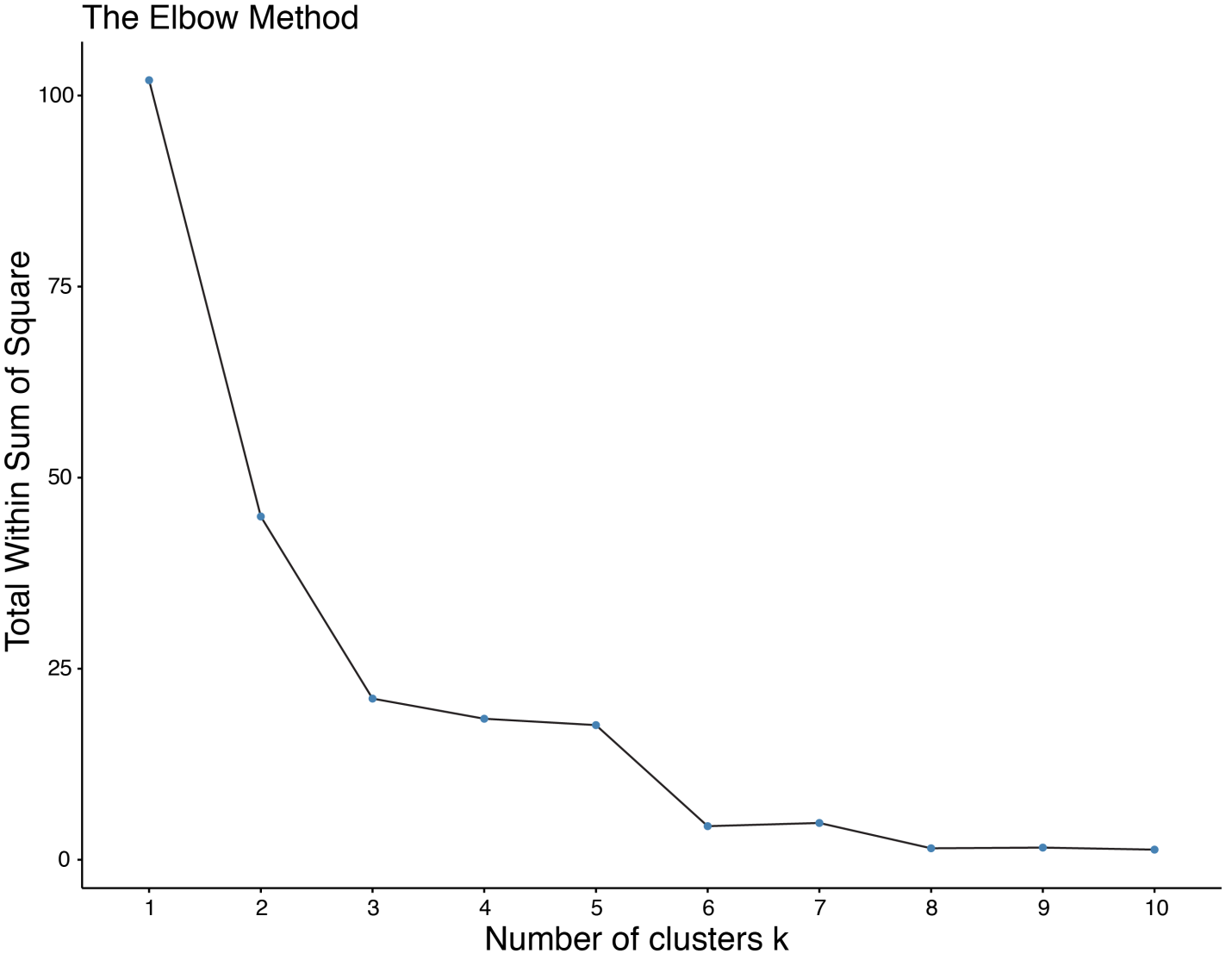


**Additional Figure S1a.** Line plot comparing total intra-cluster variation (total within sum of squares) to hypothetical number of clusters (*k*).


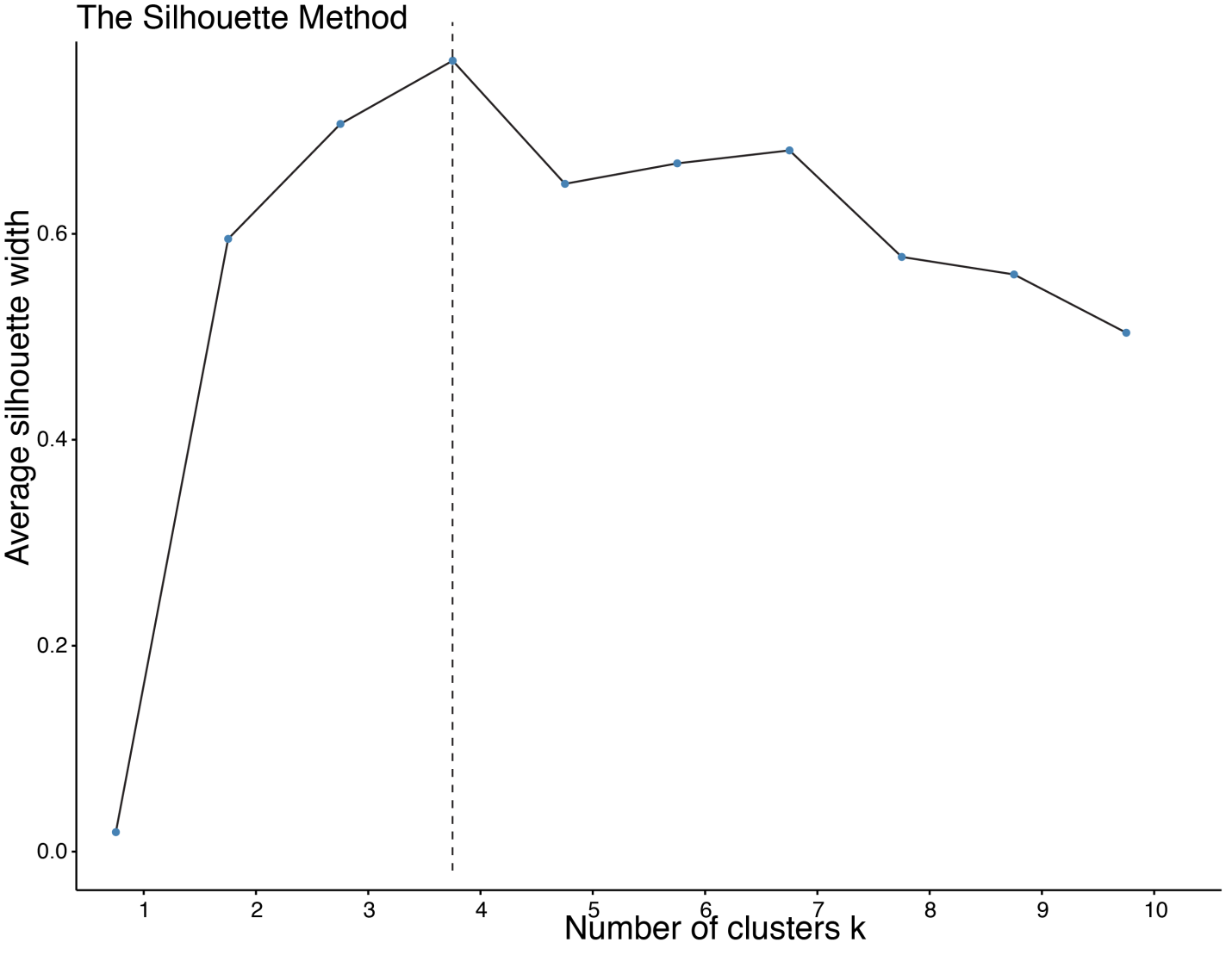


**Additional Figure S1b.** Line plot comparing average silhouette of observations for hypothetical number of clusters (*k*).


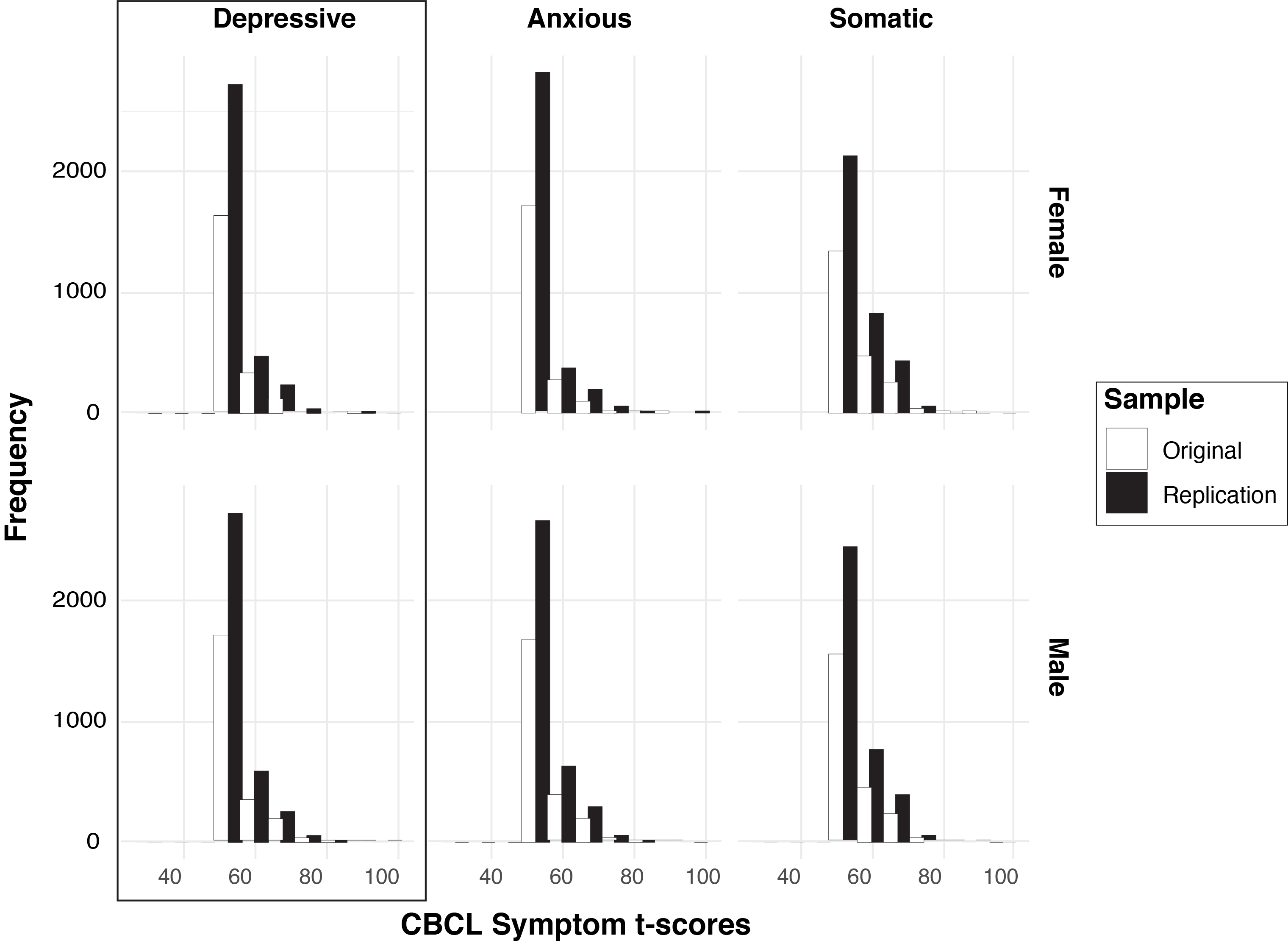
 **Additional Figure S2.** Histogram of CBCL t-scores for subscale symptoms across original (*n* = 4,393) and replication samples (*n* = 7,127).


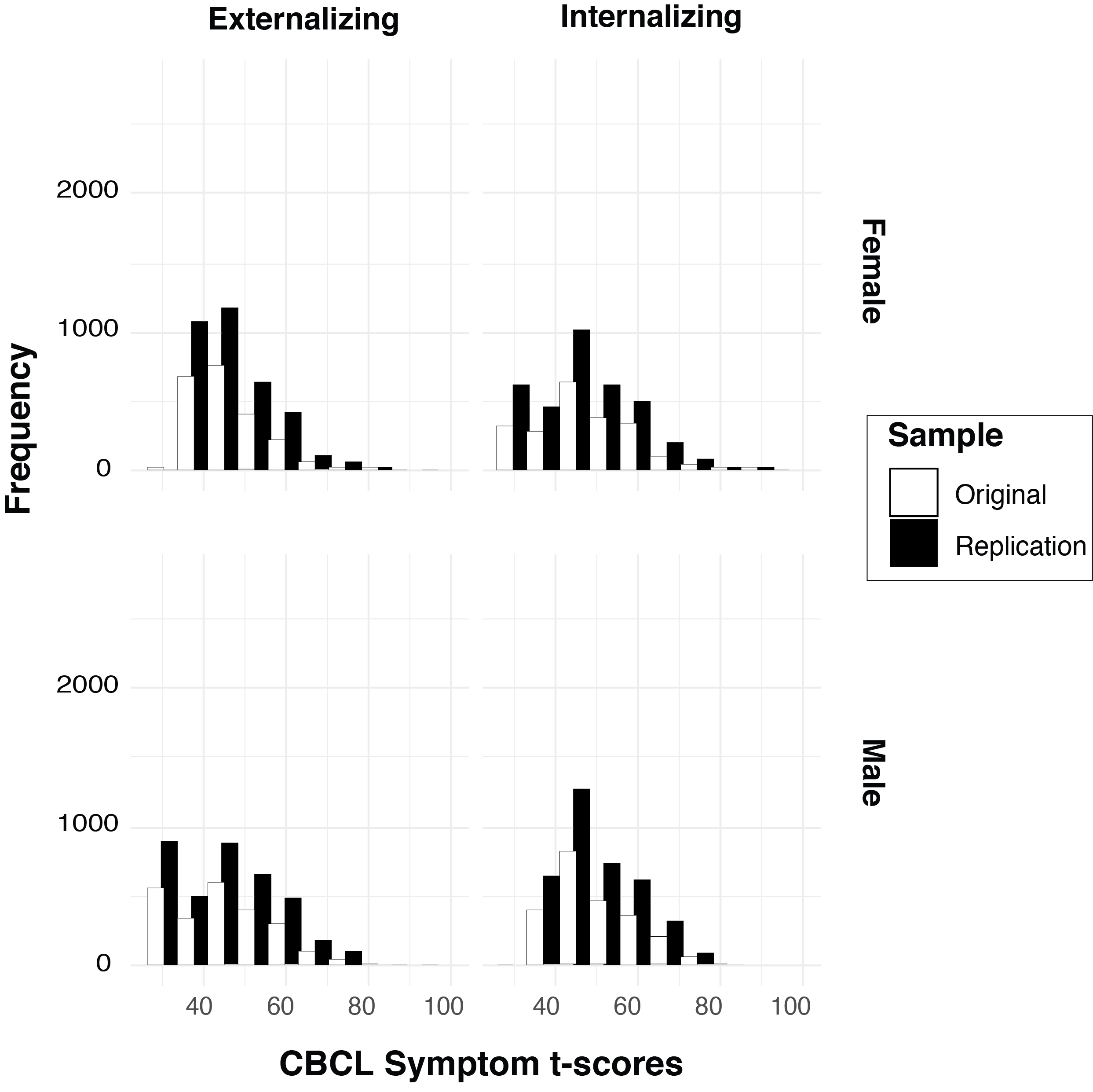


**Additional Figure S3.** Histogram of CBCL t-scores for broadband symptoms across original (*n* = 4,393) and replication samples (*n* = 7,127).

**
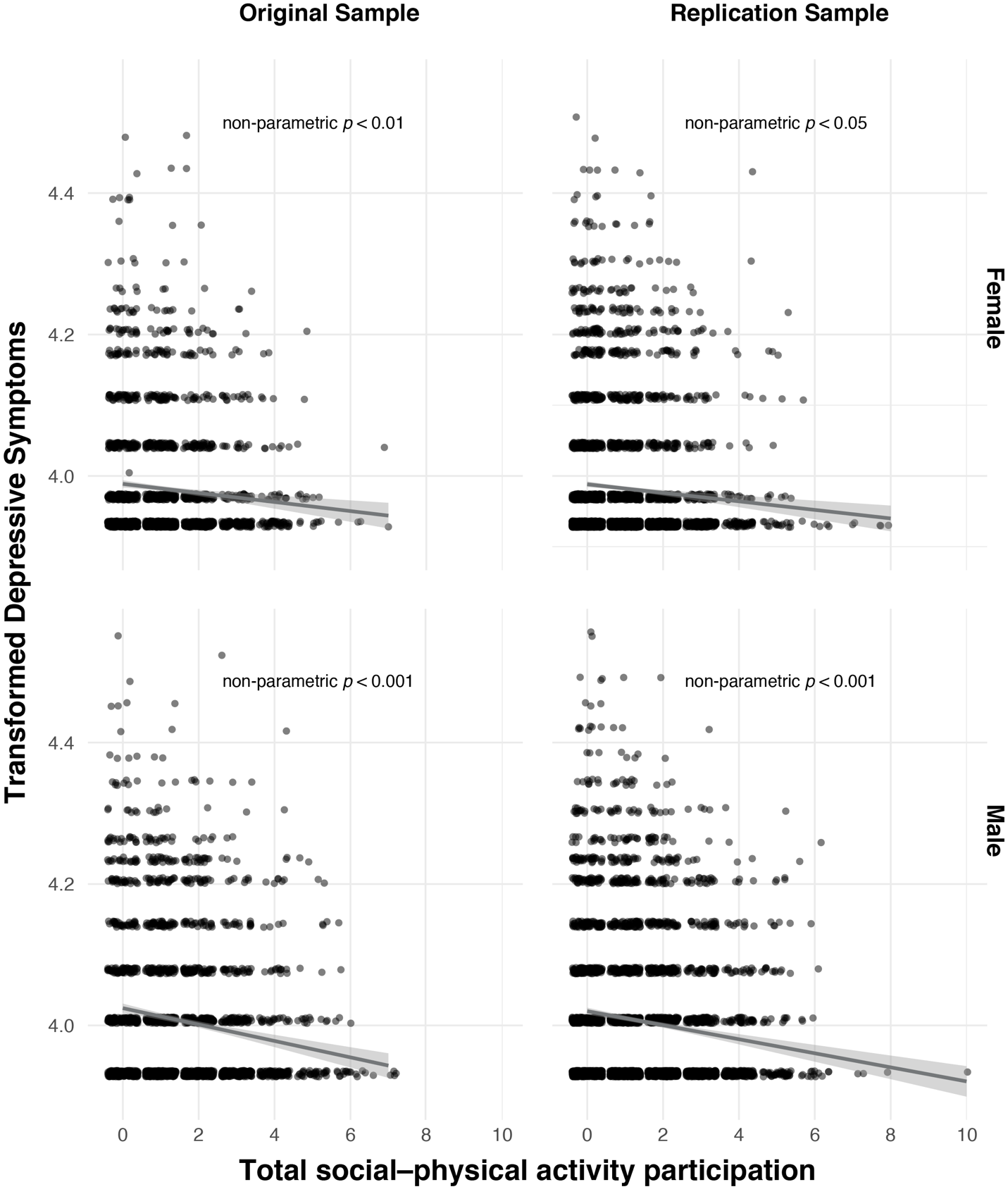
Additional Figure S4** Scatter plot of transformed depressive symptoms by participation in social–physical activities split by sex. A mixed effect model was used to examine the differences in symptoms according to activity participation in the past 12 months.

**Additional Table S1a**. Descriptive statistics for original sample internalizing t-scores across all activity categories.

|  |  | **Female** | **Male** |
| --- | --- | --- | --- |
| **Overall Internalizing** | |  |  |
|  | Mean (SD) | 47.54 (10.08) | 49.16 (10.61) |
|  | Median | 46 | 48 |
|  | IQR | 15 | 16 |
| **Anxious** | |  |  |
|  | Mean (SD) | 53.02 (5.39) | 53.66 (6.01) |
|  | Median | 51 | 50 |
|  | IQR | 4 | 7 |
| **Withdrawn/Depressive** | |  |  |
|  | Mean (SD) | 52.73 (4.86) | 54.15 (6.30) |
|  | Median | 50 | 50 |
|  | IQR | 2 | 8 |
| **Somatic** | |  |  |
|  | Mean (SD) | 55.1 (6.03) | 54.7 (6.04) |
|  | Median | 53 | 53 |
|  | IQR | 7 | 7 |
| Observations |  | 2,112 | 2,281 |

**Additional Table S1b**. Descriptive statistics for replication sample internalizing t-scores across all activity categories.

|  |  | **Female** | **Male** |
| --- | --- | --- | --- |
| **Overall Internalizing** | |  |  |
|  | Mean (SD) | 47.33 (10.64) | 49.14 (10.61) |
|  | Median | 46 | 48 |
|  | IQR | 15 | 16 |
| **Anxious** | |  |  |
|  | Mean (SD) | 53.14 (5.80) | 53.69 (5.99) |
|  | Median | 50 | 50 |
|  | IQR | 4 | 7 |
| **Withdrawn/Depressive** | |  |  |
|  | Mean (SD) | 52.79 (5.24) | 54.13 (6.25) |
|  | Median | 50 | 50 |
|  | IQR | 2 | 8 |
| **Somatic** | |  |  |
|  | Mean (SD) | 55.04 (6.08) | 54.60 (5.91) |
|  | Median | 53 | 53 |
|  | IQR | 7 | 7 |
| Observations |  | 3,469 | 3,658 |

| **Additional Table S2**. Relationship between involvement in clusters of SAIQ activities on normalized CBCL internalizing subscale symptoms. All analyses include site and family nested within site as random effects. | | | | | | |
| --- | --- | --- | --- | --- | --- | --- |
|  | **CBCL Symptom Subscales**: | | | | | |
|  | **Depressive** | | **Anxious** | | **Somatic** | |
| **Sample** | Original | Replication | Original | Replication | Original | Replication |
| **Intercept** | 3.74^***^  (0.66) | 3.98^***^  (0.51) | 3.59^***^  (0.66) | 4.39^***^  (0.53) | 4.56^***^  (0.70) | 3.10^***^  (0.54) |
| **Social–Physical** | -0.01^***^  (0.001) | -0.01^***^  (0.001) | -0.002  (0.001) | -0.003^*^  (0.001) | -0.002  (0.001) | -0.002  (0.001) |
| **Non-Social–Physical** | -0.003  (0.003) | -0.001  (0.002) | -0.005  (0.003) | -0.002  (0.002) | -0.003  (0.003) | -0.001  (0.002) |
| **Social–Non-Physical** | -0.0004  (0.002) | -0.002  (0.002) | 0.003  (0.002) | -0.0004  (0.002) | 0.002  (0.002) | 0.0003  (0.002) |
| **Non-Social–Non-Physical** | 0.01^**^  (0.002) | 0.01^***^  (0.002) | 0.004  (0.002) | 0.01^***^  (0.002) | 0.01^*^  (0.003) | 0.01^***^  (0.002) |
| **Sex** | 1.39  (0.90) | -0.34  (0.71) | 0.44  (0.91) | -0.58  (0.73) | -1.18  (0.97) | 1.49^*^  (0.74) |
| **>$100K** | -0.02^***^  (0.01) | -0.05^***^  (0.004) | -0.01  (0.01) | -0.02^***^  (0.004) | -0.02^**^  (0.01) | -0.03^***^  (0.004) |
| **$25-50K** | 0.01  (0.01) | -0.02^***^  (0.004) | 0.01  (0.01) | -0.004  (0.004) | 0.01  (0.01) | -0.01^**^  (0.005) |
| **$50-100K** | -0.01^**^  (0.01) | -0.03^***^  (0.004) | -0.001  (0.01) | -0.01^**^  (0.004) | -0.002  (0.01) | -0.02^***^  (0.004) |
| **White** | 0.01^*^  (0.005) | 0.004  (0.004) | 0.01  (0.005) | 0.004  (0.004) | 0.002  (0.005) | 0.01^**^  (0.004) |
| **Black** | -0.01^*^  (0.01) | -0.01^**^  (0.004) | -0.02^**^  (0.01) | -0.03^***^  (0.005) | -0.01^*^  (0.01) | -0.02^***^  (0.005) |
| **Asian** | -0.01  (0.01) | -0.01  (0.01) | -0.02  (0.01) | -0.02^*^  (0.01) | -0.02  (0.01) | -0.01  (0.01) |
| **Other** | 0.01  (0.01) | 0.01  (0.005) | 0.01  (0.01) | -0.003  (0.01) | 0.004  (0.01) | 0.01^*^  (0.01) |
| **Age** | 0.004  (0.01) | 0.0003  (0.01) | 0.01  (0.01) | -0.01  (0.01) | -0.01  (0.01) | 0.02  (0.01) |
| **Age^2^** | -0.0000  (0.0000) | -0.0000  (0.0000) | -0.0000  (0.0000) | 0.0000  (0.0000) | 0.0000  (0.0000) | -0.0001  (0.0000) |
| **Sex:Age** | -0.02  (0.02) | 0.01  (0.01) | -0.01  (0.02) | 0.01  (0.01) | 0.02  (0.02) | -0.03^**^  (0.01) |
| **Sex:Age^2^** | 0.0001  (0.0001) | -0.0000  (0.0001) | 0.0000  (0.0001) | -0.0000  (0.0001) | -0.0001  (0.0001) | 0.0001^**^  (0.0001) |
| **Observations** | 4,151 | 6,652 | 4,151 | 6,652 | 4,151 | 6,652 |
| **Log Likelihood** | 3,891.46 | 6,130.23 | 3,848.47 | 5,894.12 | 3,630.90 | 5,816.28 |
| **Akaike Inf. Crit.** | -7,742.91 | -12,220.47 | -7,656.95 | -11,748.23 | -7,221.79 | -11,592.56 |
| **Bayesian Inf. Crit.** | -7,616.37 | -12,084.47 | -7,530.41 | -11,612.23 | -7,095.25 | -11,456.56 |
| Note: Reporting $\beta$ (SE) *p<0.05**p<0.01***p<0.001 | | | | | | |

| **Additional Table S3**. Relationship between involvement in clusters of SAIQ activities on CBCL broadband symptoms (t-scores). All analyses include site and family nested within site as random effects. | | | | |
| --- | --- | --- | --- | --- |
|  | **CBCL Broadband Scales**: | | | |
|  |  | | | |
|  | **Internalizing** | | **Externalizing** | |
| **Sample** | Original | Replication | Original | Replication |
|  | | | | |
| **Intercept** | 62.64 (71.42) | 51.34 (55.97) | 79.06 (67.56) | 94.97 (54.21) |
|  |  |  |  |  |
| **Social–Physical** | -0.57^***^ (0.14) | -0.46^***^ (0.11) | -0.06 (0.13) | -0.12 (0.11) |
| **Non-Social–Physical** |  |  |  |  |
| **Social–Non-Physical** | -0.56 (0.30) | -0.24 (0.24) | -0.23 (0.28) | -0.24 (0.23) |
| **Non-Social–Non-Physical** |  |  |  |  |
| **Social–Physical** | 0.45 (0.25) | 0.05 (0.21) | -0.16 (0.23) | -0.14 (0.20) |
| **Non-Social–Physical** |  |  |  |  |
| **Non-Social–Non-Physical** | 0.77^***^ (0.26) | 1.21^***^ (0.22) | 0.16 (0.25) | 0.24 (0.21) |
|  |  |  |  |  |
| **Sex** | -13.48 (98.14) | -2.36 (76.72) | 4.74 (92.83) | -11.02 (74.23) |
|  |  |  |  |  |
| **>$100K** | -1.41^*^ (0.62) | -3.43^***^ (0.46) | -3.61^***^ (0.59) | -5.12^***^ (0.45) |
|  |  |  |  |  |
| **>$25-50K** | 1.27^*^ (0.66) | -1.09^*^ (0.48) | -0.90 (0.62) | -1.69^***^ (0.46) |
|  |  |  |  |  |
| **$50-100K** | 0.18 (0.60) | -2.12^***^ (0.45) | -2.23^***^ (0.57) | -3.51^***^ (0.44) |
|  |  |  |  |  |
| **White** | 0.53 (0.51) | 0.77 (0.42) | 1.16^*^ (0.48) | 1.18^**^ (0.41) |
|  |  |  |  |  |
| **Black** | -2.55^***^ (0.69) | -2.74^***^ (0.49) | 0.68 (0.66) | -0.07 (0.48) |
|  |  |  |  |  |
| **Asian** | -2.78^*^ (1.22) | -2.42^*^ (1.00) | -2.25^*^ (1.15) | -3.24^***^ (0.97) |
|  |  |  |  |  |
| **Other** | 0.68 (0.69) | 0.87 (0.53) | 1.66^**^ (0.66) | 1.86^***^ (0.52) |
|  |  |  |  |  |
| **Age** | -0.24 (1.20) | -0.09 (0.94) | -0.52 (1.13) | -0.79 (0.91) |
|  |  |  |  |  |
| **Age^2^** | 0.001 (0.01) | 0.001 (0.004) | 0.002 (0.005) | 0.003 (0.004) |
|  |  |  |  |  |
| **Sex:Age** | 0.27 (1.65) | 0.10 (1.29) | -0.03 (1.56) | 0.22 (1.25) |
|  |  |  |  |  |
| **Sex:Age^2^** | -0.001 (0.01) | -0.001 (0.01) | 0.0000 (0.01) | -0.001 (0.01) |
|  |  |  |  |  |
|  | | | | |
| **Observations** | 4,151 | 6,652 | 4,151 | 6,652 |
| **Log Likelihood** | -15,464.73 | -24,974.59 | -15,231.31 | -24,763.80 |
| **Akaike Inf. Crit.** | 30,969.45 | 49,989.19 | 30,502.63 | 49,567.61 |
| **Bayesian Inf. Crit.** | 31,095.99 | 50,125.19 | 30,629.17 | 49,703.61 |
|  | | | | |
| Note: Reporting $\beta$ (SE) *p<0.05**p<0.01***p<0.001 | | | | |

**References**

Chekroud SR, Gueorguieva R, Zheutlin AB, et al. Articles Association between physical exercise and mental health in 1·2 million individuals in the USA between 2011 and 2015: a cross-sectional study. 2018:739. doi:10.1016/S2215-0366(18)30227-X

Gorham LS, Jernigan T, Hudziak J, Barch DM. Involvement in Sports, Hippocampal Volume, and Depressive Symptoms in Children. *Biol psychiatry Cogn Neurosci neuroimaging*. 2019;0(0). doi:10.1016/j.bpsc.2019.01.011

Martel MM. Sexual selection and sex differences in the prevalence of childhood externalizing and adolescent internalizing disorders. *Psychol Bull*. 2013;139(6):1221-1259. doi:10.1037/a0032247

Nolen-Hoeksema S, Girgus JS. The emergence of gender differences in depression during adolescence. *Psychol Bull*. 1994;115(3):424-443. doi:10.1037/0033-2909.115.3.424
